# Complex Twisted Knots of Umbilical Cord in a Monochorionic-Diamniotic Twin Gestation: A Case Report

**DOI:** 10.31661/gmj.v9i0.1878

**Published:** 2020-09-16

**Authors:** Behnaz Razavi, Maryam Kasraeian, Atefe Hashemi, Shaghayegh Moradi Alamdarloo, Fateme Sadat Najib

**Affiliations:** ^1^Department of Obstetrics and Gynecology, Shiraz University of Medical Sciences, Shiraz, Iran; ^2^Maternal-Fetal Medicine Research Center, Shiraz University of Medical Sciences, Shiraz, Iran

**Keywords:** Umbilical cord Entanglement, Nuchal Cord, Multiple Entanglement, Twin Pregnancy

## Abstract

**Background:**

True knots and tight loops of umbilical cord can cause serious fetal complications in monochorionic-monoamniotic twins but are usually unexpected in Monochorionic-diamniotic twins because of the presence of the intertwin membrane. This report presents a case of monochorionic-diamniotic twin gestation with a complex cord knots.

**Case report:**

A 31-year-old G2Ab1 with monochorionic-diamniotic twin pregnancy in the gestational age of 30 weeks presented with ruptured membrane since 3weeks before delivery. At the delivery time, multiple umbilical cord knots was found.

**Conclusion:**

Premature ruptured membrane can cause septostomy of the intertwin membrane, multiple umbilical cord knots and its complications. Therefore, these cases should be considered for evaluation of the presence of intertwin membrane and umbilical cord knots in each sonography examination.

## Introduction

Monochorionic twin gestations are at a higher risk for perinatal complications such as preterm delivery, twin to twin transfusion syndrome (TTTS), intrauterine growth restriction (IUGR), and also umbilical cord knots and entanglement [1]. True knots of the umbilical cord can lead to some severe fetal complications because of the pressure on the umbilical vessels and disruption of blood flow, which can cause intrauterine growth restriction (IUGR) or fetal death [2]. This complication occurs more frequently in mono-chorionic–mono-amniotic (MCMA) twin pregnancies as a result of the rolling of the fetuses around each other. Monochorionic di-amniotic (MCDA) twins defined as two fetuses with one placental disc (or chorion) but with distinct amniotic cavities [3] are rarely at risk for complex cord knots because of the existence of an intertwin dividing membrane [1]. The current study presents the observation of complex umbilical cord knots and loops in a case of MCDA twin pregnancy with preterm rupture of membrane (PROM) and IUGR at the delivery time. The possible cause of the septostomy of the intertwin membrane in the presented case can be spontaneous rupture of the membrane.

## Case Presentation

 A 31-year-old pregnant woman gravida two abortion 1 admitted in our institution (HAZRAT ZEYNAB Hospital of Shiraz University of medical science) at gestational age (GA) of 27 + 3 weeks with the chief complaint of vaginal leakage and diagnosis of premature rupture of amniotic membrane (PROM). In her first obstetric sonography at GA of 19+2 weeks, the MCDA pattern was confirmed with adequate amniotic fluid, and no congenital anomalies were detected. On the first physical examination, no tachycardia, fever, and uterine tenderness were observed. The patient’s speculum examination revealed fern-positive vaginal leakage without malodor discharge, with a closed cervix and no uterine contraction. Ultrasound examination showed two alive fetuses, and the posterior placenta and amniotic fluid of both fetuses were adequate (5cm and 7.5cm). Strip NST of both fetuses was reactive with an FHR base of 150. Based on these examinations, the patient admitted and received IV antibiotics at first for two days, continued with oral antibiotics for seven days, and two doses of corticosteroids for lung maturation administered during 48hrs.

**Figure 1:**
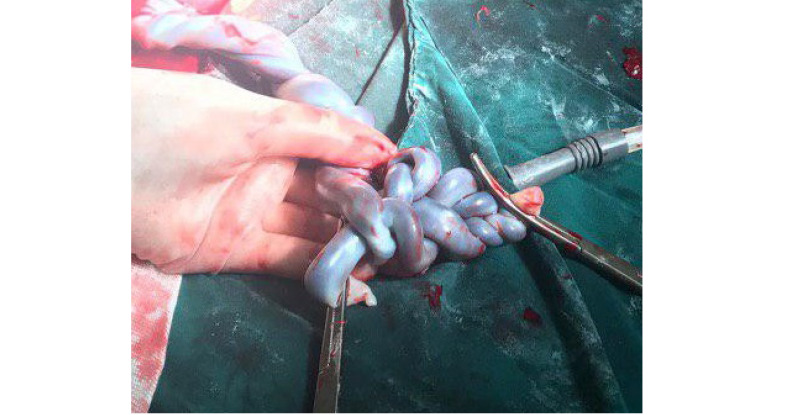
Complex true knots and tight loops of umbilical cord from two different viewpoints.

 The patient was under observation in the hospital with weekly WBC and CRP (c-reactive protein) and twice-weekly NST and maternal pulse rate (PR) and temperature monitoring for early detection of chorioamnionitis signs. In-hospital course after two weeks of admission, the biometry and colorflowmetry of the umbilical artery revealed fetal weight discrepancy of 31% and IUGR stage I for one of the fetuses and the other fetus was relatively small for gestational age (SGA). Ultrasonography also reported that no intertwin membrane was seen most probably because of the rupture of the membrane since the patient had not experienced any invasive procedure during pregnancy. Fetal heart rate monitoring was acceptable for gestational age. Also, no sign of chorioamnionitis presented until the 3rd week of admission. At the 30th week of gestation, the patient developed maternal tachycardia (PR: 110-120), fever (t: 38-38.5), a rise in WBC count up to 14000, and persistent tachycardia of the IUGR fetus (170-180). Therefore after hydration and administration of broad-spectrum antibiotics, the emergency cesarean section was performed due to chorioamnionitis diagnosis. At the time of cesarean section two female newborns delivered by breech and vertex presentation, the first one cried immediately after birth with an APGAR score of 6, weighing 1430gr and the second one born with mild respiratory depression and poor APGAR score of 1 and birth weight of 1020gr. APGAR score re-evaluation five minutes after the delivery showed the scores of 8 and 6 for the first and the second newborn, respectively. Two times nuchal cord was observed around the neck of one of the fetuses. Furthermore, two umbilical cords were twisted and formed a complex of true knots and tight loops as it is shown in Figure-1. Umbilical artery blood gas examinations showed PH: 7.4, pCo2: 35, HCO3: 21.3, base excess: -2.6, for the first fetus, and PH: 7.35, pCo2: 32, HCO3: 17.1 and base excess: -7, for the second one (the depressed baby). Both neonates were admitted in the NICU ward because of prematurity and also respiratory distress syndrome in the second twin. No significant neonatal complication occurred, except mild hyperbilirubinemia. All cultures and gram stain of throat, nose, blood, eye discharge, and CSF were negative. Brain sonography revealed grade-I IVH in the second twin. The first and the second babies were respectively discharged from NICU ward 20 and 24 days after their birth, and no complications have been observed yet in the afterbirth follow-ups. After consultation with the mother and upon her request, the placenta and umbilical cord were sent for histological examination. The anatomical pathology findings (macroscopic) confirmed the MCDA as the type of chorionicity with no specific pathological changes except cholangitis of the placenta.

## Discussion

True knots of the umbilical cord found in approximately 1% of the deliveries and can cause severe obstetric complications, such as fetal asphyxia and eventual fetal death. Predisposing factors for true umbilical cord knots include long cords, hydramnios, small fetuses, male fetuses, gestational diabetes mellitus, undergoing genetic amniocentesis, mono-amniotic twin gestations, and multiparity. Amongst these, the mono-amniotic twin gestations (and its subsequent complications) is one of the most important predisposing factors. The MCMA pregnancies have the highest perinatal mortality and morbidity rate of all twin types. One of the reasons is the highest risk of cord knots and entanglement, followed by asphyxia and fetal death in such cases. The MCDA twins, on the other hand, have an inter-twine dividing membrane and are usually not at risk for complex cord knots and entanglement. However, infrequent spontaneous septostomy of the dividing membrane in the MCDA gestations could result in a pseudo-monoamniotic environment, which in turn may cause umbilical cord entanglements, and it is complications [4]. The inter-twin membrane rupture in the MCDA twins could be caused either spontaneously or iatrogenically at the time of any invasive procedure such as amniosynthesis. In either case, the MCDA twins are then considered as pseudo-monoamniotic twins with a significantly increased risk of developing cord entanglement and increased prenatal mortality rate [5]. In a previous case series, reporting 18 monoamniotic and seven pseudo-monoamniotic cases, it is mentioned that there were no significant differences in the incidence of neonatal death between the monoamniotic and pseudo-monoamniotic twin gestations. Therefore, the same management should be considered for the monoamniotic and pseudo-monoamniotic twin gestations. In the same study of 18 monoamniotic and 7 cases of pseudo-mono-amniotic twin gestations, it is reported that the intertwin membrane rupture was caused iatrogenically in 3 cases and spontaneously in the other 4 cases. According to the results of this study, the incidence of the umbilical cord entanglement in mono-amniotic twins was 72%, and in pseudo-mono-amniotic twins was 43% with no significant difference [6]. In a case report by Sherer and colleagues, cord entanglement was found at the delivery time in an MCDA twin. The examination of the placenta confirmed an MCDA placenta with a short remnant of the intertwine membrane on the fetal side. The patient’s postoperative course was uneventful, and the mother and her newborns were discharged on the fourth postoperative day [7]. Although the determination of the exact time of intertwin membrane septostomy is not possible in our presented case, it happened most probably at the time of the rupture of the amniotic membrane at the gestational age of 27 weeks. In histopathology of the placenta, cholangitis was marked. Chorangiosis is vascular hyperplasia in terminal chorionic villi, and it usually results from longstanding and low-grade hypoxia in the placental tissue [8]. In the presented case, cholangitis can be attributed to the fetal circulation compromising by umbilical cord true knots for weeks that resulted in low-grade hypoxia. Although the septostomy of the intertwin membrane affects a small number of the MCDA pregnancies, considering the fatal complications that it can cause, we recommend dedicating the special attention of the high-risk MCDA twins. The mentioned high-risk group includes the cases who had undergone any invasive intrauterine procedures and also the cases with preterm rupture of membrane. These cases should be considered for further evaluation of the intertwin membrane in each sonography examination and umbilical cords for the possibility of umbilical cord true knot formation.

## Conclusion

 The septostomy of the intertwin membrane affects a small number of the MCDA pregnancies; however, considering its fatal complications, physicians should dedicating special attention to the high-risk MCDA twins. These cases should be considered for further evaluation of the intertwin membrane in each sonography examination. Also, umbilical cords should be evaluated for the possibility of umbilical cord true knot formation.

## Conflict of Interest

 The authors confirm that there are no known conflicts of interest associated with this publication, and there has been no significant financial support for this work that could have influenced its outcome.

## References

[1] Chmait RH, Aghajanian P, Kontopoulos EV, Quintero RA (2009 May). Prenatal diagnosis of spontaneous septostomy of the dividing membranes in complicated monochorionic diamniotic multiple gestations. J Ultrasound Med.

[2] Clerici G, Koutras I, Luzietti R, Di Renzo GC (2007). Multiple true umbilical knots: a silent risk for intrauterine growth restriction with anomalous hemodynamic pattern. FETAL DIAGN THER.

[3] Fuchs KM, D’Alton ME. Chorionicity of Multiple Gestations. InObstetric Imaging. FETAL DIAGN THER. Fetal Diagnosis and Care 2018 Jan 1 (pp. 639-641). Elsevier.

[4] Szczepanik ME, Wittich AC (2007 Aug 1). True knot of the umbilical cord: a report of 13 cases. Mil med.

[5] Lee KJ, Kim MK, Lee SY, Lee WS, Lee YH (2012 May). Spontaneous rupture of the dividing membrane in a monochorionic pregnancy resulting in a pseudo-monoamniotic pregnancy with cord entanglement. J OBSTET GYNAECOL RE.

[6] Suzuki S. Case series of monoamniotic and pseudomonoamniotic twin gestations.OBSTET GYNECOL.2013 Feb 21;2013. 10.1155/2013/369419 PMC3595717 23509635

[7] Sherer DM, Sokolovski M, Haratz-Rubinstein N (2002 Nov). Diagnosis of umbilical cord entanglement of monoamniotic twins by first-trimester color Doppler imaging. J of ultrasound med: official journal of the American Institute of Ultrasound in Medicine.

[8] Suzuki K, Itoh H, Kimura S, Sugihara K, Yaguchi C, Kobayashi Y, Hirai K, Takeuchi K, Sugimura M, Kanayama N (2009 Jun). Chorangiosis and placental oxygenation. Congenit anom.

